# Prepectoral Versus Retropectoral Implant-Based Breast Reconstruction—Considerations Regarding the Implant Pocket

**DOI:** 10.3390/jcm15156041

**Published:** 2026-08-03

**Authors:** Christine Deutschmann, Paola Clauser, Christian F. Singer, Carmen Leser, Georg Pfeiler, Daphne Gschwantler-Kaulich

**Affiliations:** 1 Department of Obstetrics and Gynecology, Division of General Gynecology and Gynecologic Oncology, Medical University of Vienna, Waehringer Guertel 18-20, 1090 Vienna, Austria; 2 Department of Biomedical Imaging and Image-Guided Therapy, Medical University of Vienna, Waehringer Guertel 18-20, 1090 Vienna, Austria

**Keywords:** prepectoral, retropectoral, implant-based breast reconstruction, implant pocket, skin and subcutaneous tissue, residual fibroglandular breast tissue, pectoralis major muscle

## Abstract

**Background/Objectives**: Prepectoral implant positioning has become an established option for implant-based breast reconstruction (IBBR) following a mastectomy. The impact of the implant position on the selected implant pocket thickness and the presence of residual fibroglandular breast tissue (RFGT) has yet to be determined. **Methods**: Patients following an IBBR between 2013 and 2017 at the Department of Obstetrics and Gynecology, Medical University of Vienna, Austria, and with an available archived breast MRI were identified through a retrospective chart review. Breast MRIs were reevaluated using Osirix. **Results**: A total of 14 patients (23 breasts) with prepectoral and 30 patients (45 breasts) with retropectoral breast implants were included. A reduction in the postoperative skin and subcutaneous tissue thickness compared to preoperative measurements was found in both cohorts, reaching statistical significance in the prepectoral subgroup (medially *p* = 0.001, laterally *p* = 0.044). Significantly higher RFGT volumes were present in the prepectoral compared to the retropectoral cohort (*p* = 0.026). This difference remained significant after accounting for unevenly distributed subgroup characteristics. In the retropectoral cohort, a significant reduction in the postoperative compared to preoperative thickness of the pectoralis major muscle (PMM) was observed (*p* < 0.001). The PMM only accounted for 30% of the implant pocket covering the breast implant. **Conclusions**: Prepectoral IBBR was associated with higher volumes of RFGT and a significant reduction in postoperative skin and subcutaneous tissue thickness compared with retropectoral reconstruction. These findings suggest that implant position may influence the quality of the implant pocket and underscore the importance of careful surgical planning and meticulous dissection. Further studies are warranted to determine the clinical significance of these findings with regard to oncologic and reconstructive outcomes. In retropectoral reconstruction, marked postoperative atrophy of the PMM was observed, raising questions about the long-term advantages of this reconstructive approach.

## 1. Introduction

Implant-based breast reconstruction (IBBR) is the predominantly performed method of breast reconstruction following a mastectomy [1]. Refined ablative techniques and the introduction of acellular dermal matrices (ADMs) and synthetic mesh products have made prepectoral implant positioning an established option in IBBR. The technique allows breast reconstruction with comparable complication rates to retropectoral implant placement while avoiding complications associated with the detachment of the pectoralis major muscle (PMM), such as animation deformity and prolonged postoperative pain [2,3,4]. The feasibility and excellent aesthetic outcomes of this technique have been demonstrated by numerous authors [5,6,7,8,9,10,11,12,13].

In the course of mastectomy, thorough preservation of the subcutaneous fat tissue layer is essential to avoid ischemic complications. A nipple-sparing mastectomy (NSM) flap thickness of less than 8.0 mm has been identified as a predictor of impaired perfusion [14].

Yet, incomplete removal of fibroglandular breast tissue (FGT) to ensure thick, well-perfused skin flaps must equally be avoided, as residual fibroglandular breast tissue (RFGT) potentially deteriorates the oncologic prognosis. RFGT is associated with the remaining occult breast cancer at the time of the ablative procedure as well as an increased local recurrence risk thereafter [15,16,17,18,19,20]. Thickness of the skin flap, type and indication of mastectomy, patient’s age and height, a recent breast surgery and breast reconstruction with a flap are associated with the amount of remaining FGT [16,21,22,23,24].

As prepectoral IBBR has become increasingly popular, studies reviewing the impact of the implant position on the selected implant pocket thickness as well as on the presence of RFGT are needed.

This is—to the best of our knowledge—the first study addressing this highly topical research question. Furthermore, the role of the PMM in shaping the implant pocket in retropectoral IBBR will be studied.

## 2. Materials and Methods

Patients following an IBBR between 2013 and 2017 at the Department of Obstetrics and Gynecology, Medical University of Vienna, Austria, and with an available archived breast MRI at least one year following the reconstructive procedure were identified through a retrospective chart review. Patients with second-look resections, flap reconstructions, and fat grafting procedures prior to the postoperative breast MRI or no implant in place at the time of the MRI were excluded.

**Figure 1:**
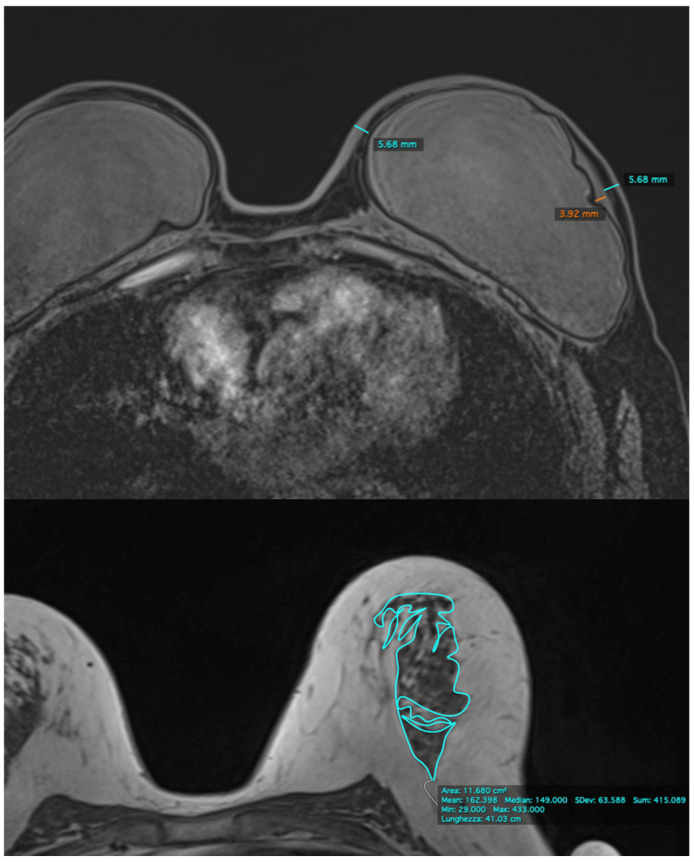
Measurement of the thickness of skin and subcutaneous fat tissue medially and laterally (green), and of RFGT laterally (orange, **upper** figure). Measurement of FGT volume (green, **lower** Figure). RFGT, residual fibroglandular breast tissue, and FGT, fibroglandular tissue.

**Figure 2:**
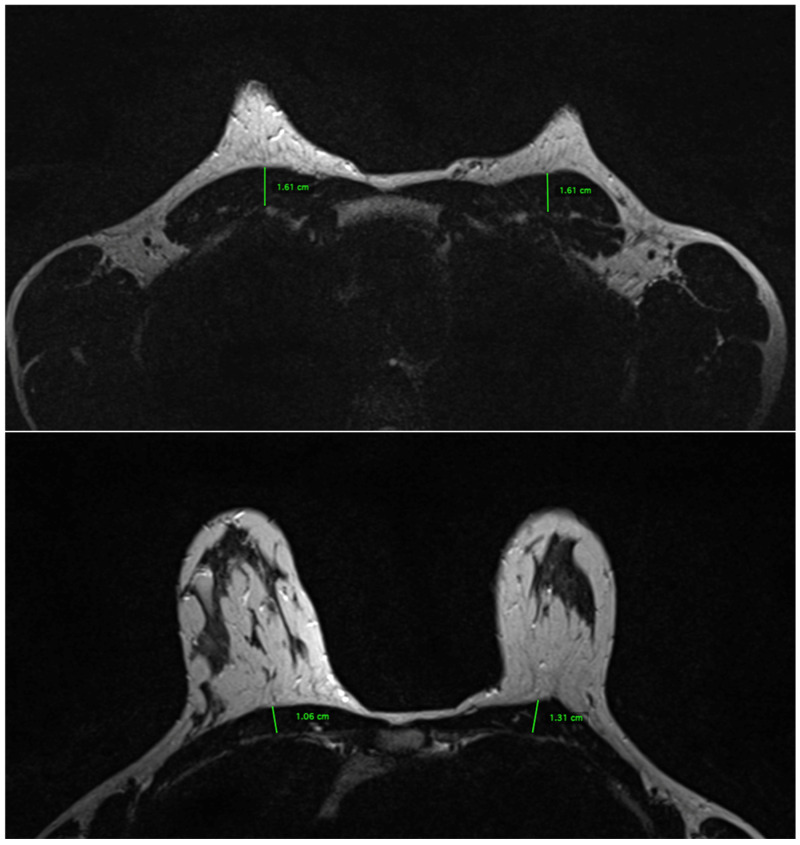
Measurement of the thickness of the PMM measured centrally at the level of the manubrium sterni (green, **upper** figure) and at the level of the cranial breast quadrant (green, **lower** figure); PMM, pectoralis major muscle.

**Figure 3:**
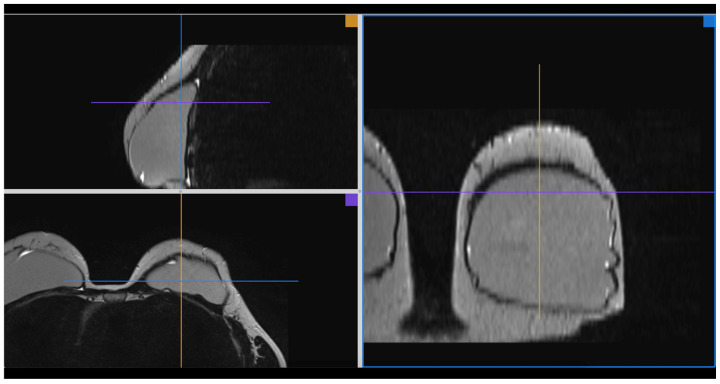
Measurement of the percentage of the PMM covering the implant surface; PMM, pectoralis major muscle.

Postoperative and—if available—preoperative breast MRIs were reevaluated using Osirix regarding the following parameters: 1. pre- and postoperative skin and subcutaneous fat tissue thickness measured at the level of the nipple medially and laterally (see Figure 1); 2. the volume of FGT preoperatively (see Figure 1); 3. the thickness of RFGT measured at the level of the nipple medially, laterally and retroareolarly (see Figure 1); 4. the volume of RFGT and—solely in the retropectoral cohort—5. the pre- and postoperative thickness of the PMM measured centrally at the level of the manubrium sterni and at the level of the cranial breast quadrant (see Figure 2); and 6. the percentage of implant surface covered by the PMM (see Figure 3).

All MRI measurements were performed by one board-certified radiologist with extensive experience in breast MRI. Blinding to the reconstruction type was not feasible, as the implant position was readily identifiable on MRI and could not be concealed without interfering with the measurements.

The 44 patients were categorized into two groups: prepectoral cohort and retropectoral cohort. Each affected breast was considered independent regardless of whether it originated from the same patient. Given the retrospective exploratory nature of the study, no formal sample size or power calculation was performed. Descriptive statistics were performed for all 44 patients and are provided in counts and percentages, mean and 95% CI, or median with corresponding ranges. A *t*-test was used to evaluate differences in mean between prepectoral and retropectoral groups for continuous variables, and the Mann–Whitney test was used when the values were not normally distributed. Chi-square or Fisher’s test was used to compare categorical data. For all analyses, a *p*-value < 0.05 was considered statistically significant. SPSS statistical software system (SPSS Inc., Chicago, IL, USA, version 23.0) was used for all calculations.

## 3. Results

### 3.1. Demographics

In total, 14 patients (23 breasts) with prepectoral IBBR and 30 patients (45 breasts) with retropectoral IBBR met the inclusion criteria and were enrolled in the study.

Demographic data were well balanced between the two cohorts except for age, BMI, radiotherapy (RT), and the use of an ADM or synthetic mesh. The mean age in the prepectoral cohort was lower than in the retropectoral subgroup (36.7 years (30.8–42.6) versus 43.2 years (38.6–47.9)). The mean BMI was lower in the retropectoral compared to the prepectoral subgroup (18.3 kg/m^2^ versus 22.1 kg/m^2^). In six cases of the retropectoral cohort, RT was performed, compared to none of the cases in the prepectoral cohort. The majority of cases in the prepectoral cohort (65.2%) were reconstructed with an ADM compared to the predominant use of a mesh in the retropectoral cohort (48.9%). Ten cases in the retropectoral cohort received neither ADM nor a synthetic mesh.

Further demographic details are outlined in Table 1.

### 3.2. Implant Pocket

A reduction in the postoperative compared to preoperative skin and subcutaneous fat tissue thickness was observed in both cohorts, reaching statistical significance in the prepectoral subgroup (medially *p* = 0.001 and laterally *p* = 0.044).

RFGT was found in all breasts in both cohorts. Notably, in one breast in the retropectoral cohort, volumetry was not feasible; therefore, this breast was excluded from the analysis. Statistically higher RFGT volumes were present in the prepectoral compared to the retropectoral subgroup (*p* = 0.026). This difference remained significant after accounting for unevenly distributed subgroup characteristics (see Table 2). Furthermore, RT and skin-sparing mastectomy (SSM) vs. nipple-sparing mastectomy (NSM) did show a statistically significant association with RFGT volume in the multiple regression analysis, with lower volumes of RFGT in patients who received RT and SSM, respectively.

In the retropectoral cohort, a statistically significant reduction in the postoperative compared to the preoperative PMM thickness measured at the level of the manubrium sterni and at the level of the cranial breast quadrant was observed (*p* < 0.001).

The PMM only accounted for 30% of the implant pocket covering the breast implant.

Further details regarding the implant pocket are displayed in Table 3.

## 4. Discussion

Prepectoral positioning of the breast implant has become an established surgical approach in IBBR. The present study aimed to evaluate the impact of the implant position on the implant pocket. Pre- and postoperative MRI images of patients following mastectomy and prepectoral versus retropectoral IBBR were reevaluated using Osirix.

The study found a reduction in the postoperative skin and subcutaneous fat tissue thickness compared to preoperative measurements in both cohorts, reaching statistical significance in the prepectoral subgroup (medially *p* = 0.001, laterally *p* = 0.044). Generally, recommendations regarding the optimal skin envelope thickness diverge between 2 and 10 mm [25,26,27]. Larson et al. [28] evaluated the subcutaneous tissue thickness in breast specimens following reduction mammoplasties. The dermal thickness ranged between 0.3 cm and 1.0 cm and the subcutaneous tissue between 0.0 cm and 2.9 cm. Furthermore, differences in the thickness of the subcutaneous tissue were shown between both breasts within one patient. Concordantly to the present study, Frey JD et al. [14] evaluated pre- and postoperative MRIs following NSMs and found the average overall postoperative NSM flap thickness to be significantly less than preoperatively. NSM flap thickness of less than 8.0 mm was identified as an independent predictor of ischemic complications. Roy De Vita et al. [29] evaluated over 2000 NSMs and found a statistically significant association between complications and skin flaps of less than 5 mm. Furthermore, over time, hypoxic conditions in the skin flap might also increase the risk of capsular fibrosis and reconstruction failure, as shown previously [30,31]. Hence, it has to be noted that FGT resection in the course of mastectomy should be performed in the patients’ individual dissection plane, assuring thorough preservation of the skin and subcutaneous fat tissue thickness in order to avoid surgical complications.

In addition to thorough preservation of the skin flap, complete removal of FGT is aspired to in the course of mastectomy. Yet, high prevalence rates of RFGT following mastectomy have been addressed by numerous authors [21,22,23]. Woitek et al. [24], for example, detected RFGT in 20% of breasts following NSM or SSM and significantly more frequently in patients with NSM than SSM. Histopathological studies on skin flaps that would remain after SSM of 42 breast cancer patients showed residual breast tissue in approximately 60% of the cases and even residual disease in 9.5% [16]. Giannotti DG et al. [21] analyzed over 500 postmastectomy-reconstructed breast magnetic resonance imaging examinations and found RFGT in almost 30% of the cases. In the present study, RFGT was found in all breasts of both cohorts. None of the cases were associated with residual disease. The comparatively high prevalence of RFGT in the present study can be explained by the stringent mode of measurement, the performance of volumetry in comparison to solely the length measurements, and the use of MRIs for RFGT assessment.

A significant difference in RFGT volume was found between the two operative methods, with higher volumes of RFGT being present in the prepectoral cohort (*p* = 0.026). Several baseline characteristics differed between the two cohorts and may have influenced the observed differences in RFGT volume. The prepectoral cohort comprised younger patients (median 36.7 years vs. 43.2 years) with a higher BMI (median 22.1 vs. 18.3 kg/m^2^), both known risk factors for RFGT [32,33]. Furthermore, the prepectoral cohort consisted of a higher percentage of patients with neoadjuvant chemotherapy (21.4% vs. 10%), possibly impeding the definition of the correct dissection plane in the course of mastectomy owing to changes in the tissue architecture following treatment. The prepectoral cohort also contained a higher percentage of NSMs (78.3% vs. 57.7%) and breast reconstructions with an ADM (100% of reconstructed breasts vs. 74.5%), which have been associated with higher rates of RFGT in the literature [32]. Additionally, a higher percentage of immediate primary fixed-volume implants (100% vs. 88.9%) was noted in the prepectoral cohort, potentially leading to higher volumes of RFGT owing to the attempt to create a thick, well-vascularized skin flap in the course of mastectomy owing to the lack of the PMM for implant coverage [32]. Yet, the difference in RFGT volume between the prepectoral and retropectoral cohorts remained statistically significant after adjustment for these baseline differences in a multiple regression analysis (*p* = 0.043). In addition, RT and SSM (vs. NSM) were independently associated with lower RFGT volumes in the multiple regression analysis, with lower volumes of RFGT in patients who received RT and SSM (compared to NSM), respectively.

The thickness of RFGT showed a statistically significant correlation with the postoperative skin- and subcutaneous fat tissue thickness measured medially: *p* < 0.001 and laterally: *p* = 0.001. This is in accordance with Torresan RZ et al. [16], who showed that the presence of terminal ductal lobular units in skin flaps that would remain after SSM was significantly associated with skin flaps thicker than 5 mm. Giannotti DG et al. [21] analyzed over 500 postmastectomy-reconstructed breast magnetic resonance imaging examinations for RFGT and also found the thickness of the flap to be associated with the presence of RFGT. On the other hand, Woitek R et al. [24] found no association between the presence of RFGT and the flap thickness.

Furthermore, the study showed that disinsertion and subsequent atrophy of the PMM in the course of retropectoral implant placement led to a statistically significant reduction in muscle thickness (*p* < 0.001). Postoperatively, the PMM only represented 30% of the implant pocket covering the breast implant. Previous authors demonstrated that, particularly, postmastectomy radiation therapy promoted scarring of the PMM [30]. Based on these findings and the known sequelae of muscle disinsertion in the course of retropectoral implant placement, including disruption of muscle function, animation deformities, and prolonged postoperative pain [34,35,36], the PMM should be questioned regarding whether it is adequate for implant coverage.

The present study has several limitations. Its retrospective single-center design may have introduced information and selection bias and may limit the generalizability of the findings. The small sample size, the imbalance between the two cohorts, and the absence of a formal sample size calculation limited the statistical power of the study and may have affected the precision of the estimates. Therefore, the findings should be considered exploratory and require confirmation in larger prospective studies before definitive conclusions can be drawn. Bilateral breasts were analyzed as independent observations, although measurements from the same patient may not be fully independent. All MRI measurements were performed by a single radiologist, and interobserver reproducibility was not evaluated. Furthermore, although oncologic outcomes, including local recurrence and metastatic disease, were assessed, the low event rate precluded statistical evaluation of the association between implant position and oncologic outcomes. Finally, the choice of implant position was at the discretion of the operating surgeon and may have been influenced by patient characteristics, surgeon experience, and the gradual adoption of prepectoral reconstruction following technical advances, including the introduction of ADMs and synthetic meshes. While retropectoral reconstruction represented the standard approach at the beginning of the study period, prepectoral reconstruction became increasingly established over time, introducing the potential for selection bias.

## 5. Conclusions

Prepectoral IBBR was associated with higher volumes of RFGT and reduced postoperative skin and subcutaneous tissue thickness compared with retropectoral reconstruction. These findings suggest that implant position may influence both the quality of the implant pocket and the amount of RFGT following mastectomy and highlight the importance of careful surgical planning and meticulous dissection. In retropectoral reconstruction, substantial postoperative atrophy of the PMM was observed, raising questions about the long-term advantages of this reconstructive approach. Future studies are needed to determine the clinical implications of these findings with regard to oncologic outcomes, surgical complication rates, and reconstructive results.

## Figures and Tables

**Table 1:** Demographic data.

Demographic Data	Prepectoral Cohort	Retropectoral Cohort
**No. patients/breasts**	14/23	30/45
**Age** (mean years, 95% CI)_PP_	36.7 (30.8–42.6)	43.2 (38.6–47.9)
**BMI** (median kg/m^2^)_PP_	22.1	18.3
**Neoadjuvant chemotherapy**_PP_	6	4
**Radiotherapy**_PB_	0	6
**Mastectomy type**_PB_		
- Simple mastectomy	0	4
- Nipple-sparing mastectomy	18	26
- Skin-sparing mastectomy	5	15
**Indication for mastectomy**_PP_		
- Oncologic	6	17
- Prophylactic	12	22
**Time of reconstruction and type of prosthesis**_PB_		
- Immediate, primary fixed-volume implant	23	40
- Immediate, primary tissue expander	0	6
- Delayed, primary tissue expander	0	1
**ADM and synthetic mesh**_PB_		
- ADM	15	13
- Synthetic mesh	8	22
- None	0	10
**Time between preoperative and postoperative MRI** (median months, range)_PP_	13 (11–24)	25.5 (11–46)
**Oncologic outcome**_PP_ *		
- Local recurrence	1	3
- Metastatic disease	1	1
**Follow-Up** (mean months)_PP_ *	23.0 (±14.7)	50.4 (±24.0)

PP: analysis per patient, PB: analysis per breast; * only cases with oncologic mastectomy indication considered.

**Table 2:** Multiple regression analysis and associations of demographic parameters with RFGT.

Multiple Regression Analysis	β	T	SIG.
Implant position	−0.288	−2.090	0.043
Radiotherapy	−0.435	−2.485	0.017
Skin-sparing mastectomy	−0.418	−2.842	0.007
Simple mastectomy	−0.231	−0.950	0.348
Age	0.160	0.983	0.331
BMI	0.031	0.239	0.812
Neoadjuvant chemotherapy	0.003	0.019	0.985
Indication Cancer	−0.029	−0.183	0.855
Indication HBOC	−0.177	−1.025	0.311
Time of reconstruction and type of prosthesis	0.032	0.139	0.890
Surgeon	0.110	0.775	0.443

BMI: Body mass index, HBOC: Hereditary breast and ovarian cancer syndrome.

**Table 3:** Implant pocket.

Implant Pocket	Prepectoral Cohort	Retropectoral Cohort	*p*-Value
**Skin and subcutaneous fat tissue**			
- Preoperative			
- Medial (median mm, range)	9.0 (4.0–13.0)	7.0 (3.0–16.0)	0.084
- Lateral (median mm, range)	8.0 (2.0–12.0)	6.0 (4.0–18.0)	0.43
- Postoperative			
- Medial (median mm, range)	4.0 (1.0–10.0)	5.0 (1.0–20.0)	0.007
- Lateral (median mm, range)	5.0 (1.0–10.0)	7.0 (1.0–15.0)	0.118
**Fibroglandular tissue**			
- Volume (median cm^3^, range)	64.1 (20.0–106.0)	70.2 (6.4–286.1)	0.992
**Residual fibroglandular tissue**			
- Medial (median mm, range)	0 (0–1)	0 (0–6)	0.027
- Lateral (median mm, range)	0 (0–3)	0 (0–10)	0.902
- Retroareolar (median mm, range)	3.50 (0–21)	1.0 (0–15)	0.073
- Volume (median cm^3^, range)	4.0 (0.8–17.8)	1.9 (0–15.5)	0.026
**Pectoralis major muscle**			
- Preoperative			
- Manubrium sterni (median mm, range)		9.0 (1.2–17.0)	
- Cranial quadrant (median mm, range)		7.0 (4.0–9.0)	
- Postoperative			
- Manubrium sterni (median mm, range)		7.0 (2.0–10.0)	
- Cranial quadrant (median mm, range)		2.0 (1.0–6.0)	
- Implant coverage (% median, range)		30.0 (5.0–40.0)	

All variables were analyzed per breast.

## Data Availability

Data are unavailable for distribution due to privacy restrictions.

## Abbreviations

The following abbreviations are used in this manuscript:

ADM	Acellular dermal matrix
CI	Confidence interval
FGT	Fibroglandular breast tissue
HBOC	Hereditary breast and ovarian cancer syndrome
IBBR	Implant-based breast reconstruction
NSM	Nipple-sparing mastectomy
PB	Analysis per breast
PMM	Pectoralis major muscle
PP	Analysis per patient
RFGT	Residual fibroglandular breast tissue
RT	Radiotherapy
SSM	Skin-sparing mastectomy
